# “Please mind the gap”: successful use of ultrasound-assisted spinal anesthesia for urgent cesarean section in a patient with implanted spinal cord stimulation system for giant chest wall arteriovenous malformation – a case report

**DOI:** 10.1186/s12871-020-01042-6

**Published:** 2020-05-23

**Authors:** Bruno Antonio Zanfini, Salvatore De Martino, Luciano Frassanito, Stefano Catarci, Francesco Vitale di Maio, Pietro Paolo Giuri, Gian Luigi Gonnella, Gaetano Draisci

**Affiliations:** Fondazione Policlinico Universitario Agostino Gemelli IRCCS, Università Cattolica del Sacro Cuore, Scienze dell’Emergenza, Anestesiologiche e della Rianimazione, Largo A. Gemelli 8, 00168 Rome, Italy

**Keywords:** Spinal cord stimulation (SCS) system, Chest wall arteriovenous malformations, Cesarean section, Ultrasound

## Abstract

**Background:**

The use of Spinal Cord Stimulation (SCS) system to treat medically refractory neuropathic pain is increasing. Severe neuropathic pain can be found in giant chest wall arteriovenous malformations (AVMs), exceedingly rare and debilitating abnormalities, rarely reported during pregnancy.

**Case presentation:**

We present a report of a pregnant patient with implanted Spinal Cord Stimulation (SCS) system because of painful thoracic AVM scheduled for an urgent cesarean section in which we used lumbar ultrasound (US) to rule out the possibility to damage SCS electrodes and to find a safe site to perform spinal anesthesia.

**Conclusions:**

The use of lumbar US to find a safe site for a lumbar puncture in presence of SCS system in a patient affected by painful thoracic AVM makes this case a particularly unique operative challenge and offers a new possible use of ultrasound to detect a safe space in patients with SCS implant.

## Background

The use of Spinal Cord Stimulation (SCS) system to treat medically refractory neuropathic pain is increasing [1]. Severe neuropathic pain can be found in giant chest wall arteriovenous malformations.

Arteriovenous malformations (AVMs) are rare abnormalities, associated with other congenital syndromes (Rendu-Osler-Weber Syndrome) or consequence of trauma, infection, cancer. Congenital AVMs can be found usually in the central nervous system but can be reported in abdominal organs (liver and gastrointestinal tract), thoracic organs (lung and heart) and lower limb. Congenital chest wall AVMs are rare, with few case reports available in the literature [2–6], but extremely debilitating: if no treatment is performed thoracic AVMs can cause severe bleeding, cardiac failure associated with arteriovenous shunting and severe neuropathic thoracic pain.

We report a case of a 29 years old pregnant patient with a giant, congenital, painful AVM of the left chest wall, treated with implanted Spinal Cord Stimulation (SCS) system, admitted to our obstetric emergency room for an urgent cesarean section.

The presence of SCS system in pregnant patients with congenital AVM and the use of lumbar ultrasound (US) to perform a safe spinal anesthesia made this case a particularly unique operative challenge.

## Case presentation

**Fig. 1:**
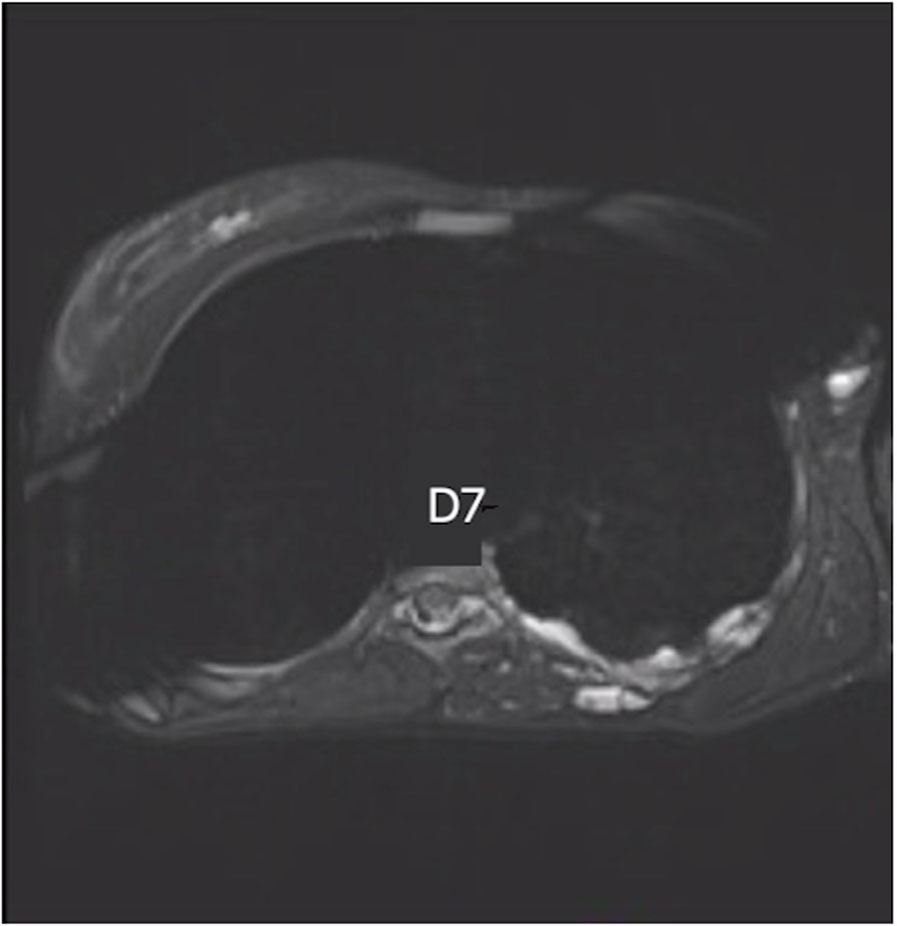
MRI axial view

**Fig. 2:**
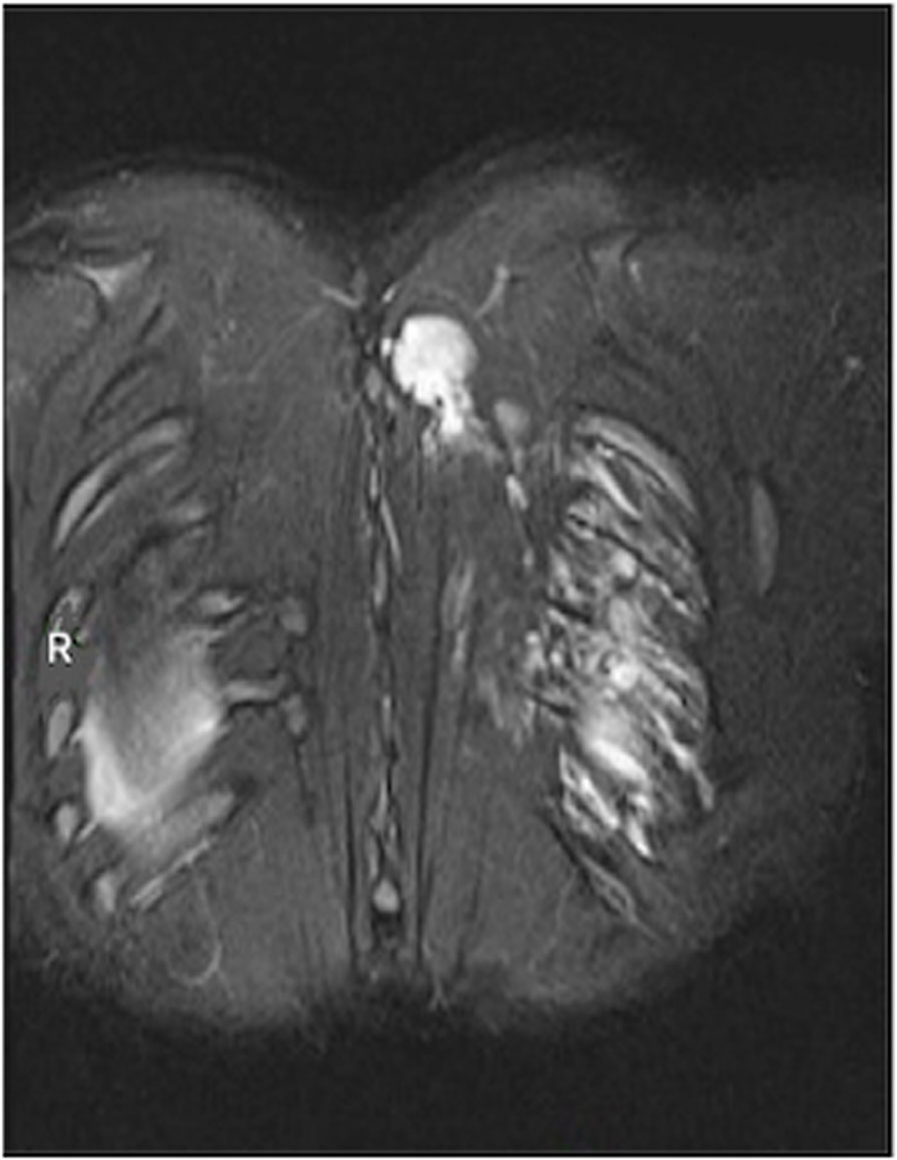
MRI coronal view

**Fig. 3:**
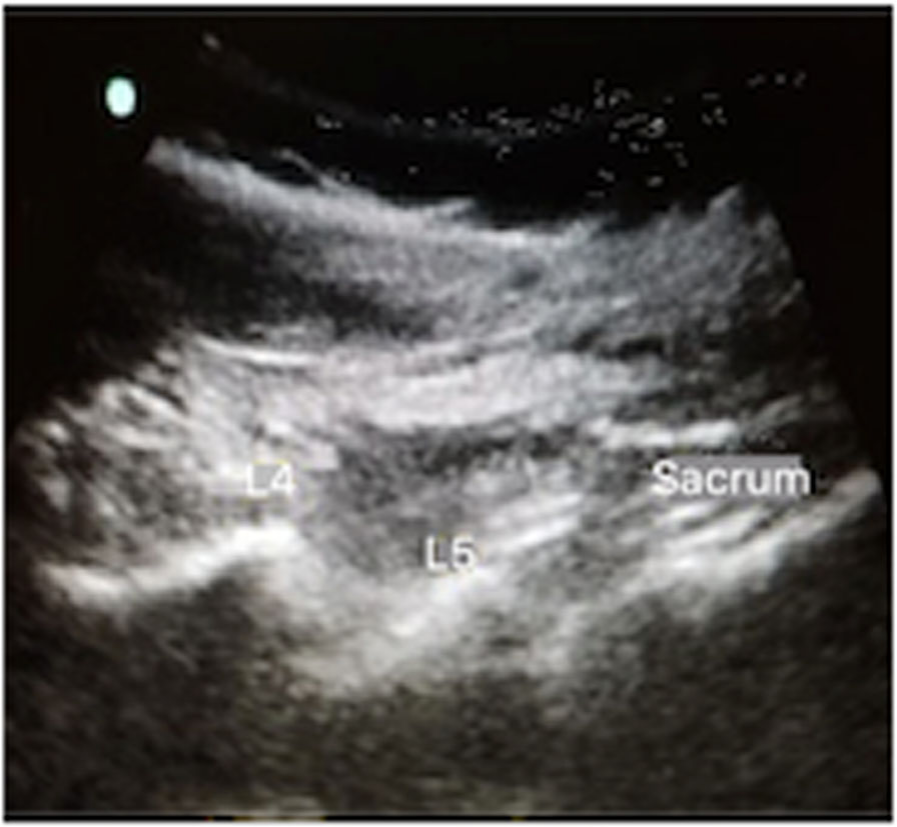
Parasagittal oblique view of lumbar spine

**Fig. 4:**
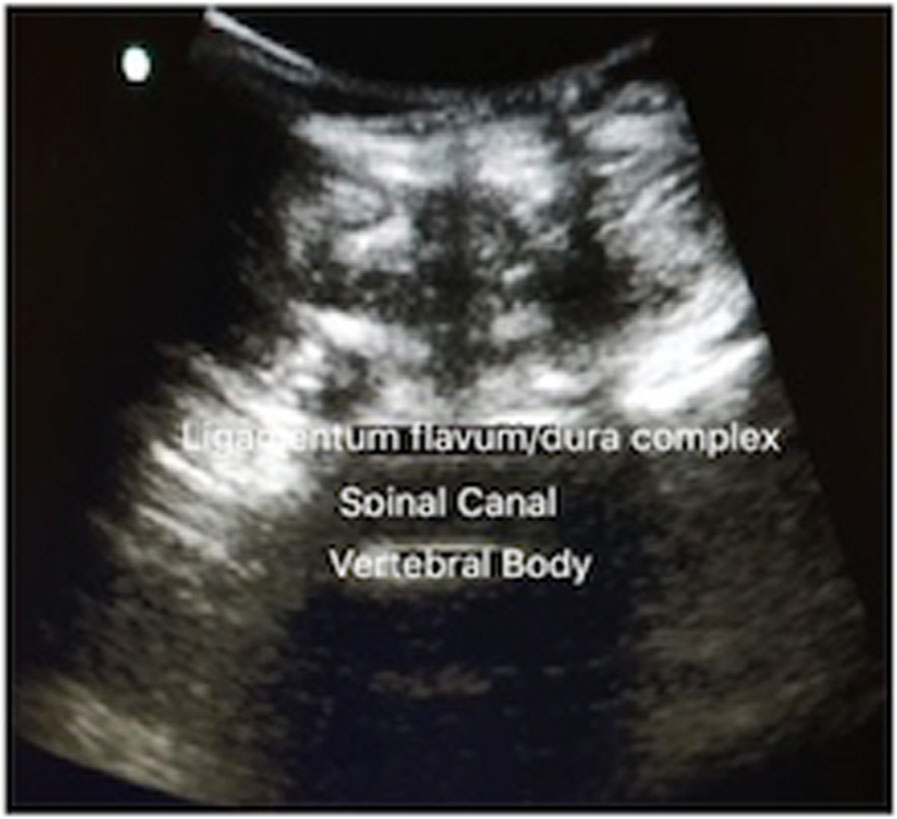
Transverse interlaminar view at L4-L5 interspace

**Fig. 5:**
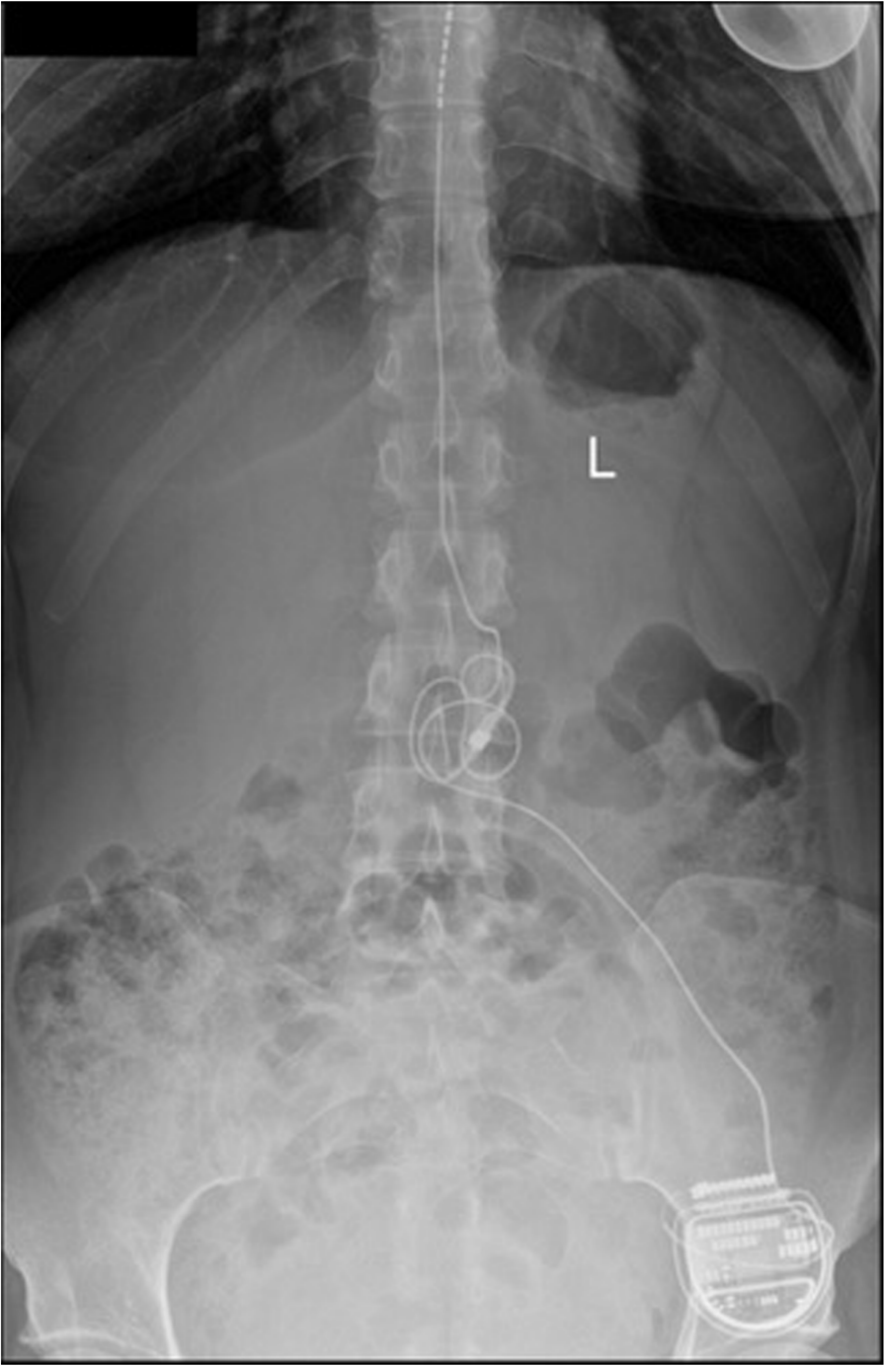
Antero-posterior X ray of lumbar and thoracic spine

**Fig. 6:**
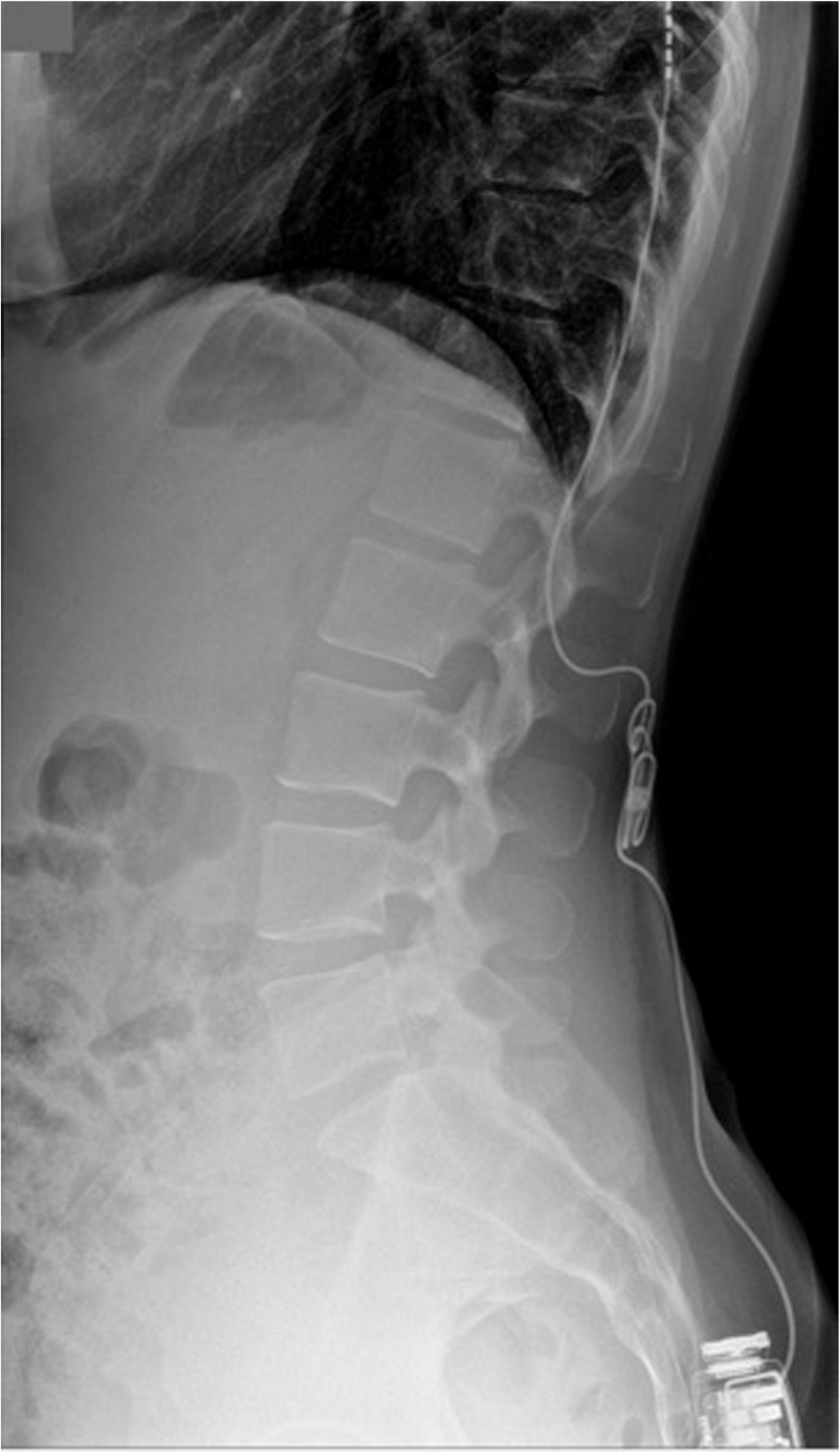
Lateral X ray of lumbar and thoracic spine

A 29 years old pregnant patient with singleton pregnancy at 38th gestational week (GW) was admitted to our obstetric emergency room for membrane rupture. She referred to be affected by a giant, congenital AVM of the left chest wall, extending from the third to the seventh thoracic interspace, involving the overlying thoracic muscles and the correspondent thoracic nerves, the rib cage, the parietal pleura, the transverse process of seventh thoracic vertebra and the scapular girdle, with severe neuropathic pain and reduced mobility in her left arm. She underwent to multiple embolizations to treat the lesion, with no or mild improvement of her pain despite she was taking 60 mg Oral Morphine Equivalents (OME) per day and pregabalin 300 mg twice daily. To treat her medically refractory neuropathic pain she was therefore referred for SCS system implant. A Model SC-1200 Precision™ Montage™ Magnetic Resonance Imaging (Boston Scientific) was successfully implanted, with electrodes at thoracic level and Implantable Pulse Generator (IPG) in the left buttock. The procedure led to a dramatic improvement of the symptoms and withdrawal of opioids and antiepileptic drugs in 8 weeks. One year later she obtained a spontaneous pregnancy. During pregnancy her pain control improved, leading to SCS deactivation through the period. Despite the absence of stimulation she reported her neuropathic pain “go into remission”. So she needed no other medications. Her pregnancy progressed normally until 38th GW. At admission just a pre-SCS implant Magnetic Resonance Imaging (MRI) was available (Fig. 1, axial view; Fig. 2, coronal view) thus making unpredictable the location of her SCS leads, extensions, and IPG. An urgent cesarean section was planned because of the membrane rupture and the risk of severe bleeding due to a possible AVM rupture in case of vaginal birth, due to the increasing of thoracic pressure during pushing (urgent CS type 3 according to NICE classification) [7]. To rule out a possible lesion to SCS system and to identify a safe lumbar interspace, a US assisted spinal anesthesia has been performed. No drugs have been administered before surgery. During anesthesiologic and surgical procedure standard hemodynamic monitoring has been provided for pregnant [Continuous Electrocardiographic monitoring (ECG), Pulse oximetry (SpO2), Non-invasive Blood Pressure (NIBP)]; fetal wellbeing has been registered by cardiotocographic monitoring. Supplemental oxygen has been provided by Venturi mask. In sitting position, after skin disinfection with surgical solution (ChloraPrep®, Carefusion, 244 LTD, UK) and using a broadband (5–8 MHz) convex probe, a left US paramedian sagittal oblique view has been obtained, starting at the sacrum and moving cephalad, to identify the L4-L5 lumbar interspaces (Fig. 3, paramedian sagittal oblique view). To identify the neuraxial midline, after rotating the probe 90° into a transverse orientation, a transverse interlaminar view has been obtained (Fig. 4, transverse interlaminar view). No electrodes have been reported in that interspace. After local anesthesia with lidocaine 2% (5 mL) a 25-gauge Whitacre spinal needle has been used to perform a spinal anesthesia using hyperbaric bupivacaine 0.5% 10 mg plus sufentanil 5 mcg and morphine 100 mcg intrathecally administered. Surgical procedure started when T4 level has been reached. An uneventful cesarean section has been performed. A healthy 3395-g female baby was born (Apgar scores of 9–10 at both 1 and 5 min). A postoperative thoracic X-Ray confirmed the right placement of thoracic electrodes but with entry point of SCS leads at L2-L3 interspace (Fig. 5, antero-posterior X ray; Fig. 6, lateral X ray). At postoperative day 3 patient has been discharged home.

## Discussion and conclusions

Our case presents the successful use of lumbar US to detect a safe space for intrathecal injection in a patient with SCS implant because of thoracic AVM. Current data suggest that landmark identification using a pre-procedure ultrasound (US) is a useful adjunct to neuraxial anesthesia [8–10] that facilitates technical performance in obstetric [11–13] and pediatric patients. In adult patients with difficult spinal anatomy [14, 15] a pre-procedure US reduces the number of attempts [16] and the number of needle passes necessary for successful spinal anesthesia [16] and can predict technical difficulty [14, 17]; notably, compared to fluoroscopy, sonography allows for the elimination of radiation exposure for physician and for patient, mostly when, as in our case during a urgent admission to obstetric emergency room, a X-ray scan to identify the location of SCS leads, extensions, and IPG cannot be performed. Even though the first description of a SCS implant for pain in pregnant patients occurred only in 1999 [18] the number of pregnant patients with SCS is increasing. Mostly of these pregnants are affected by Complex Regional Pain Syndrome (CPRS) or Failed Back surgery Syndrome (FBSS) [19–24] with a SCS system implanted before pregnancy occurred. Because leads migration is one of the most common complications occurring in from 2.1 to 27% out of 5000 patients undergoing SCS [25], we performed a lumbar ultrasound scan to identify a safe interspace where perform a spinal anesthesia. The safest choice of anesthesia for cesarean section in pregnant patients with a vascular malformation requires careful consideration. No data have been reported about thoracic AVMs to pregnancy; most of data can be derived from AVMs of Central Nervous System (CNS). Ong discussed the relative risks of different anesthetic choices for cesarean for a patient with a known cervical (C3) AVM that was stable throughout pregnancy [26]. Although general anesthesia can provide good hemodynamic stability, airway manipulation may lead to coughing and bucking with attendant increases in intrathoracic and venous pressures on waking from the anesthesia. This has potential to precipitate rupture of the cervical AVM. We supposed the same for thoracic AVMs and this is the reason why we choose a spinal anesthesia.

A critical decision for anesthetic management of patients with implanted SCS system for painful thoracic AVM scheduled for urgent cesarean section is the feasibility of providing a safe neuraxial anesthesia. Potential risks include damage to electrodes with the spinal or epidural needle, introducer, or catheter. Ultrasound examination may be useful to rule out these risks.

## Data Availability

All data generated or analyzed during this study are included in this published article.

## Abbreviations

AVMs: Arteriovenous malformations
SCS: Spinal Cord Stimulation
US: Ultrasound
OME: Oral Morphine Equivalents
IPG: Implantable Pulse Generator
ECG: Continuous Electrocardiographic monitoring
SpO2: Pulse oximetry
NIBP: Non-invasive Blood Pressure
MHz: MegaHertz
CPRS: Complex Regional Pain Syndrome
EMF: Electromagnetic force
CNS: Central Nervous System
